# Disrupted Patterns of Rich-Club and Diverse-Club Organizations in Subjective Cognitive Decline and Amnestic Mild Cognitive Impairment

**DOI:** 10.3389/fnins.2020.575652

**Published:** 2020-10-15

**Authors:** Chen Xue, Haiting Sun, Guanjie Hu, Wenzhang Qi, Yingying Yue, Jiang Rao, Wenjie Yang, Chaoyong Xiao, Jiu Chen

**Affiliations:** ^1^Department of Radiology, The Affiliated Brain Hospital of Nanjing Medical University, Nanjing, China; ^2^Department of Pediatrics, Xijing Hospital, The Fourth Military Medical University (Air Force Medical University), Xi’an, China; ^3^Institute of Brain Functional Imaging, Nanjing Medical University, Nanjing, China; ^4^Department of Psychosomatics and Psychiatry, ZhongDa Hospital, School of Medicine, Southeast University, Nanjing, China; ^5^Department of Rehabilitation, The Affiliated Brain Hospital of Nanjing Medical University, Nanjing, China; ^6^Institute of Neuropsychiatry, The Affiliated Brain Hospital of Nanjing Medical University, Fourth Clinical College of Nanjing Medical University, Nanjing, China

**Keywords:** amnestic mild cognitive impairment, subjective cognitive decline, rich club, diverse club, graph theory

## Abstract

**Background:**

Subjective cognitive decline (SCD) and amnestic mild cognitive impairment (aMCI) were considered to be a continuum of Alzheimer’s disease (AD) spectrum. The abnormal topological architecture and rich-club organization in the brain functional network can reveal the pathology of the AD spectrum. However, few studies have explored the disrupted patterns of diverse club organizations and the combination of rich- and diverse-club organizations in SCD and aMCI.

**Methods:**

We collected resting-state functional magnetic resonance imaging data of 19 SCDs, 29 aMCIs, and 28 healthy controls (HCs) from the Alzheimer’s Disease Neuroimaging Initiative. Graph theory analysis was used to analyze the network metrics and rich- and diverse-club organizations simultaneously.

**Results:**

Compared with HC, the aMCI group showed altered small-world and network efficiency, whereas the SCD group remained relatively stable. The aMCI group showed reduced rich-club connectivity compared with the HC. In addition, the aMCI group showed significantly increased feeder connectivity and decreased local connectivity of the diverse club compared with the SCD group. The overlapping nodes of the rich club and diverse club showed a significant difference in nodal efficiency and shortest path length (*L*_p_) between groups. Notably, the *L*_p_ values of overlapping nodes in the SCD and aMCI groups were significantly associated with episodic memory.

**Conclusion:**

The present study demonstrates that the network properties of SCD and aMCI have varying degrees of damage. The combination of the rich club and the diverse club can provide a novel insight into the pathological mechanism of the AD spectrum. The altered patterns in overlapping nodes might be potential biomarkers in the diagnosis of the AD spectrum.

## Introduction

Subjective cognitive decline (SCD) refers to memory complaints subjectively with a normal performance at objective neurocognitive assessments (Jessen et al., 2014a,b). SCD is considered to be an earlier stage of amnestic mild cognitive impairment (aMCI) and has a high predictive value for Alzheimer’s disease (AD) (Slot et al., 2019). aMCI, characterized by memory decline, is believed to be the intermediate stage between normal aging and dementia (Albert et al., 2011; Sperling et al., 2011). Some population-based prospective studies showed that patients with aMCI have a higher risk of developing AD. This might mean that SCD and aMCI could be regarded to be preclinical AD spectrum and may have distinct pathological characteristics in different stages of the AD spectrum (Xue et al., 2019). Furthermore, previous studies have indicated that the preclinical AD spectrum might show commonality or specificity in neuroimaging features, and these features were closely related to clinical features, such as neurocognition. Therefore, comparing and analyzing the neuroimaging characteristics of SCD and aMCI may allow deep-going prediction of the disease development and give insight into mechanisms of AD spectrum-related cognitive dysfunction.

In recent years, resting-state functional magnetic resonance imaging (rs-fMRI) and graph theory methods have become important means of cognitive research and provided a topological perspective on human brain networks (Wang et al., 2013). Sporns et al. (2005) proposed the conception of the human connectome, which declared that the human brain could be formed as a network. There are many important properties in the brain network, which is fundamentally important in cognitive neuroscience, such as small-world characteristic (Van Den Heuvel et al., 2008; Iturria-Medina et al., 2011; Wang et al., 2013; Shu et al., 2018), global and local network efficiency (Gong and He, 2015; Shu et al., 2018), and rich-club organization (Colizza et al., 2006; Sporns et al., 2007; Gong and He, 2015). The “rich club” is formed by high-degree nodes in the brain network, which tend to be more closely connected among themselves than with the lower-degree nodes (Van Den Heuvel and Sporns, 2011; Daianu et al., 2014). It is thought to be critical for information integration and communication among brain regions (Yan et al., 2018). Recent researches proposed that “diverse club” formed by a high participation coefficient has edges diversely distributed throughout the network (Bertolero et al., 2017). Some evidence suggested that the diverse club is more highly interconnected and displays stronger clubness than the rich club (Bertolero et al., 2017). Additionally, Sheffield et al. reported that the participation coefficient assesses the importance of the node to inter-network communication, whereas the degree instead measures the connection of the node within or between networks (Guimera and Amaral, 2005a; Sheffield et al., 2016). Therefore, the rich and diverse clubs play distinct roles in network communication, among other connectivity metrics, which may reflect disease pathology and contribute to revealing the distinct and unique pattern of the brain’s connectome (Daianu et al., 2015; Bertolero et al., 2017).

Several neuroimaging studies have indicated that cognitive decline in the AD spectrum is not merely caused by the damage of a single region or network (Shu et al., 2018). Actually, the global and local efficiencies of the whole brain have altered in the AD spectrum, in addition, the small-world characteristic and nodal and modular network metrics (Shu et al., 2012; Li et al., 2018). Moreover, research showed that hubs, i.e., rich-club nodes, appear to be vulnerable and before impact in AD patients (Albert et al., 2000; Shu et al., 2018). Compared with healthy controls (HCs), the number of rich-club nodes was remarkably decreased in the AD patients (Lee et al., 2018; Dai et al., 2019). The normalized rich-club coefficient of AD was higher than that of HC, which can be an important biomarker of AD (Daianu et al., 2013). Additionally, the rich-club connection strength already decreased in the SCD group, and the distinct disrupted topological phenomenon of brain connectome can be beneficial to distinguishing the aMCI converters from non-converters (Sun et al., 2018). Pusil et al. (2019) applied functional connectivity to magnetoencephalographic (MEG) data and found network failure in progressive MCI. Moreover, they suggested that stable MCI and progressive MCI both had characteristic changes in functional connectivity, which can be important features to study the evolution of AD and predict the probability of transformation. However, very little research has been done on the diverse-club organization in AD-related diseases. The participation coefficient was already used as one of the means of verifying the aging of the brain functional network in other neuropsychiatric diseases (Sheffield et al., 2016). A thorough and comprehensive understanding of the topological organization, especially rich club and diverse club, may deepen the understanding of the pathological process in the AD spectrum (Deletoile and Adeli, 2017; Dai et al., 2019).

In the current study, we used rs-fMRI to construct individual whole-brain functional networks and combined with graph theory approaches to research the topological alteration in the SCD and aMCI. The objective of the present study was to reveal the changing patterns of the rich-club and diverse-club organizations as the disease progresses. Moreover, we further analyzed the nodal metrics of overlapping nodes in the rich club and diverse club to dig the clinical significance in progress in the AD spectrum. We hypothesized that the patterns of the rich-club and diverse-club changes are not completely consistent in disease progression, and the altered patterns of nodal metrics in overlapping nodes may conduce to a different level of cognitive decline across the preclinical AD spectrum.

## Materials and Methods

### Participants

Data were obtained from the public database of Alzheimer’s Disease Neuroimaging Initiative (ADNI).^1^ We have selected 76 data (19 SCDs, 29 aMCIs, and 28 HCs) from the second phase of ADNI (ADNI-2 studies), who had undergone structural scans, rs-fMRI scans, and neurocognitive assessment. The details regarding the diagnostic criteria of the SCD, aMCI, and HC groups were provided in Supplementary Methods.

### Ethics Approval and Consent to Participate

Ethical approval for the ADNI study was provided by the institutional review committees of all participating institutions. All participants or authorized representatives offered written informed consent (see footnote 1).

### MRI Scanning

All MRI data were gained, applying a 3.0 Tesla Philips scanner. The details regarding image acquisition parameters (structure images and rs-fMRI images) were provided in Supplementary Methods.

### Image Preprocessing

The preprocessing was conducted by MATLAB2013b^2^ and RESTplus,^3^ which was based on the Statistical Parametric Mapping software package (SPM12^4^). The details regarding image preprocessing were provided in Supplementary Methods.

### Network Construction

In the current study, we constructed functional networks from rs-fMRI images with the following procedures. Networks consist of nodes and edges, where nodes represent the units of the network, i.e., brain regions, and edges represent connections between those regions, i.e., connections between brain regions. We used the Automated Anatomical Labeling template to distribute the brain into 90 regions. Pearson’s correlation coefficients between the time series of each pair of regions of interest were calculated by GRETNA software^5^ based on MATLAB. As a result, we obtained 90 × 90 connectivity matrix of each subject. The matrix was then binarized using a wide range of sparsity values (from 5 to 50%, step = 0.05) for all network analyses (Kim et al., 2014; Prajapati and Emerson, 2020). To increase the reliability of the result, the algorithm was set as 1,000 times in the following analyses.

### Network Properties

Recent researches have suggested that small-world topology exists in functional brain networks (Van Den Heuvel et al., 2008; Sun et al., 2014). To research the topologic attribute of a network, the study assessed the following graph metrics (see Supplementary Methods): characteristic path length (*L*_p_), normalized characteristic path length (λ), clustering coefficient (*C*_p_), normalized clustering coefficient (γ), small-world parameters (σ), global efficiency (*E*_g_), and local efficiency (*E*_loc_). Additionally, for each property, we calculated the area under the curve, which provides a scalar independent of threshold selection to characterize the topological characteristics of the brain network.

### Rich-Club Organization

Rich-club regions were defined as the top 13 (15%) regions with the highest node degree (i.e., the number of edges connected to the node) averaged of the HC group (Daianu et al., 2015; Yan et al., 2018). Except for the rich-club regions, other regions were defined as peripheral nodes. In addition, the edges were divided into three types of connections: rich-club connections, connecting two rich-club nodes; feeder connections, connecting one rich node and one peripheral node; and local connections, connecting two peripheral nodes (Van Den Heuvel et al., 2013). The connectivity strength was a summary measure of connectivity, which was composed of the sum of edge weights for each connection type (Yan et al., 2018).

### Diverse-Club Organization

Diverse-club regions were defined as the top 13 (15%) regions with the highest participation coefficient averaged of the HC group. The definition of participation coefficient is the diversity of connectivity of a node across communities in the network (Guimera and Amaral, 2005a; Bertolero et al., 2017). The participation coefficient is maximal when a node has an equal number of edges to each community (Bertolero et al., 2017; Bailey et al., 2018). Following the classification of the nodes into diverse-club regions and other regions, the edges in the network were also categorized into diverse-club, diverse-feeder, and diverse-local connections.

### Overlapping Nodes in Rich- and Diverse-Club Organizations

Overlapping nodes were defined as the nodes both in the rich-club and diverse-club organizations. The overlapping nodes with high node degree and high participation coefficient were the key nodes in the functional network. To explore the role of the overlapping nodes in the functional network and disease progression, we analyzed the disruption of node metrics in overlapping nodes between three groups, including the nodal degree, nodal efficiency, nodal clustering coefficient, nodal shortest path length, and betweenness centrality. Furthermore, we analyzed the alteration of average node metric in overlapping nodes, rich-club regions except for the overlapping nodes, and diverse-club regions except for the overlapping nodes between three groups. In this way, we may reveal the changing pattern of overlapping nodes as the disease spectrum progresses.

### Statistical Analysis

The Statistical Package for Social Science software version 22.0^6^ was used for all statistical analyses. The multimodal general linear model, analysis of variance (ANOVA), and the chi-square test were used to test for group differences in the demographic and neurocognitive data. The Bonferroni correction was used for *post hoc* comparisons with a *p*-value < 0.05.

One-way ANOVA was performed to compare the difference in network topology metrics, nodal topology metrics, and connectivity strength across three groups, including SCD, aMCI, and HC. All the results were earned after controlling for the effects of age and sex. The two-sample *T*-test (age- and sex-corrected) was applied for *post hoc* analyses (*p* < 0.05, Bonferroni-corrected).

The partial correlation analyses were conducted to assess how the topologic metrics related to clinical performance, including Mini-Mental State Exam (MMSE), Geriatric Depression Scale (GDS), the Wechsler Logical Memory Immediate (LMT), and LMT Delayed Recall (LMT-Delayed) in the groups after controlling for the effects of age and sex (*p* < 0.05, Bonferroni-corrected).

### Hippocampal Volume Analysis

To further explore the structural transformation process of the preclinical AD spectrum, the current study calculated the hippocampal volumes of the three groups, including the aMCI, SCD, and HC. The three-dimensional T1-weighted images were preprocessed for voxel-based morphometry analyses using the Data Processing and Analysis of Brain Imaging (Yan et al., 2016) implemented in MATLAB2013b (see footnote 2) with the following preprocessing steps: Spatial normalization was performed to create a study-specific template so that all images could be registered in the same stereotactic space. MRIs were segmented into gray, white matter, and cerebrospinal fluid images and spatially registered to the Montreal Neurological Institute and Hospital coordinate system using the DARTEL toolbox. Images were smoothed with a spatial filter with a Gaussian kernel (full width at half maximum = 8 mm). The resolution of the resulting GMV images was 1.5 mm× 1.5 mm× 1.5 mm.

After creating the mask of the bilateral hippocampus from the Wake Forest University PickAtlas toolbox (version 3.0.4,^7^ Advanced Neuroscience Imaging Research Laboratory, Wake Forest University School of Medicine, Winston-Salem, NC, United States), we extracted the averaged volume of the bilateral hippocampus in three groups and conducted a statistical analysis in Statistical Package for Social Science software. All the results were earned after controlling for the effects of age and sex. The two-sample *T*-test (age- and sex-corrected) was applied for *post hoc* analyses (*p* < 0.05, Bonferroni-corrected).

## Results

### Demographic and Neurocognitive Characteristics

Demographic and neurocognitive information of all patients consisting of 29 aMCIs (mean age 69.86 ± 7.18 years), 19 SCDs (71.95 ± 5.09 years), and 28 HCs (72.66 ± 4.42 years) are shown in Table 1. There were no meaningful group differences in age and sex (*p* > 0.05). Group differences (age and sex covariates) were observed in cognitive representation, including MMSE, GDS, and LMT-immediate and LMT-delayed. The SCD and aMCI groups exhibited significantly higher scores in GDS compared with the HC. The aMCI group showed significantly lower LMT-immediate and LMT-delayed scores compared with the SCD and HC groups (all *p* < 0.05).

**TABLE 1:** Demographics and clinical measures of HC and patients with SCD and aMCI.

	**aMCI (29)**	**SCD (19)**	**HC (28)**	***F*-values (χ^2^)**	***P*-values**
Age (years)	69.86 (7.18)	71.95 (5.09)	72.66 (4.42)	1.779	0.176
Sex (F/M)	10/19	10/9	20/8	0.211	0.646
MMSE	27.97 (1.59)	28.95 (1.43)	28.82 (1.42)	3.363	0.04
GDS	1.38 (1.01)**	1.16 (1.07)*	0.39 (0.88)	7.739	0.001^ab^
LMT-immediate	7.66 (3.15)***^/^***	14.32 (3.48)	14.72 (2.34)	43.95	<0.001^ac^
LMT-delayed	5.24 (2.37)***^/^***	13.10 (3.51)	13.46 (4.83)	74.238	<0.001^ac^

Numbers are given as means (standard deviation) unless stated otherwise. Scores reflect the number of correct items unless stated otherwise. Values for age derived from ANOVA; sex from chi-square test; all clinical measures from ANOVA with age and sex as covariates. MMSE, Mini-Mental State Examination; GDS, Geriatric Depression Scale; LMT, Wechsler Logical Memory. ^*a*^*Post hoc* analyses showed a significant group difference between aMCI and HC. ^*b*^*Post hoc* analyses showed a significant group difference between SCD and HC. ^*c*^*Post hoc* analyses showed a significant group difference between aMCI and SCD. *p < 0.05; **p < 0.01; ***p < 0.001. aMCI, amnestic mild cognitive impairment; SCD, subjective cognitive decline; HC, healthy control.

### Network Topological Metrics

The network topological metrics of three groups (HC, SCD, and aMCI) are shown in Table 2. Over a wide range of sparsity (5–50%), all the groups showed small-world attributes with a larger clustering coefficient (γ >> 1) and similar characteristic path length (λ ≈ 1). The results of ANOVA in the area under the curve of network topological metrics are shown in Table 2. Group differences (ANOVA; age and sex covariates) were found in the normalized clustering coefficient (γ) (*F* = 3.672, *p* = 0.030) and characteristic path length (*L*_p_) (*F* = 3.568, *P* = 0.033). Compared with the HC, the aMCI group showed lower γ and higher *L*_p_. Additionally, there were significant group differences in small-worldness (σ) (*F* = 4.498, *p* = 0.014) and network global efficiency (*E*_g_) (*F* = 4.706, *p* = 0.012). Compared with the HC, a significant reduction was seen in the aMCI group on small-worldness (*p* = 0.022) and global efficiency (*p* = 0.011) (Bonferroni-corrected). There was no significant difference in network topological properties between the SCD patients and the HC group.

**TABLE 2:** Comparison of network topology difference among SCD, aMCI, and HC.

**Network topological metrics**	**aMCI**	**SCD**	**HC**	***F*-values**	***P*-values**
Clustering coefficient (*C*_p_)	0.265 (0.004)	0.267 (0.005)	0.259 (0.004)	1.005	0.371
Normalized clustering coefficient (γ)	0.992 (0.017)*	1.006 (0.020)	1.056 (0.017)	3.672	0.030^a^
Characteristic path length (*L*_p_)	0.934 (0.015)*	0.917 (0.018)	0.874 (0.016)	3.568	0.033^a^
Normalized characteristic path length (λ)	0.508 (0.005)	0.510 (0.006)	0.500 (0.005)	0.910	0.407
Small-worldness (σ)	0.863 (0.014)*	0.868 (0.016)	0.919 (0.014)	4.498	0.014^a^
Global efficiency (*E*_g_)	0.253 (0.002)*	0.255 (0.002)	0.260 (0.002)	4.706	0.012^a^
Local efficiency (*E*_loc_)	0.339 (0.002)	0.342 (0.002)	0.340 (0.002)	0.356	0.702

Numbers are given as means (standard deviation) unless stated otherwise. ^*a*^*Post hoc* analyses showed a significant group difference between aMCI and HC. *p < 0.05. aMCI, amnestic mild cognitive impairment; SCD, subjective cognitive decline; HC, healthy control. All the results from ANOVA with age and sex as covariates. Bonferroni correction was used for *post hoc* comparisons with p-value < 0.05.

### Disrupted Rich-Club and Diverse-Club Organizations in Subjective Cognitive Decline and Amnestic Mild Cognitive Impairment

**FIGURE 1:**
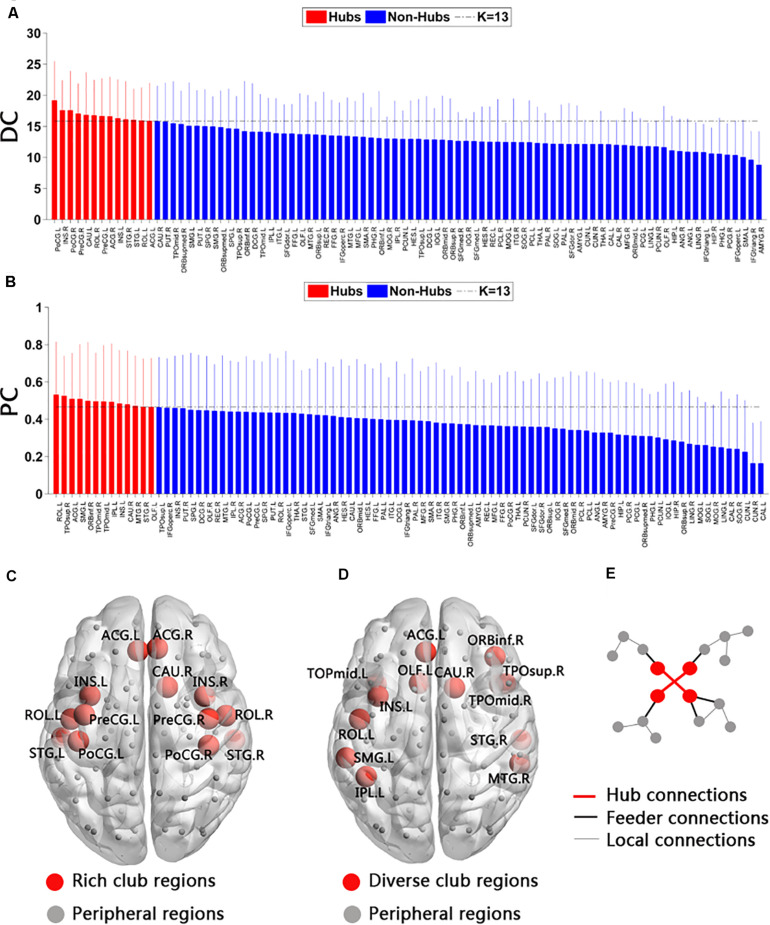
Rich-club and diverse-club organizations of functional networks. **(A)** Order was determined according to the node degree in averaged functional brain network at sparsity (S) = 15% in the healthy control (HC) group. Red represents rich-club regions (the top 13 regions), and blue represents peripheral regions. **(B)** Order was determined according to the participation coefficient in average functional brain network at S = 15% in the HC group. Red represents diverse-club regions (the top 13 regions), and blue represents peripheral regions. DC, degree centrality; PC, participation coefficient. **(C)** Rich-club regions (red nodes) of HC groups. **(D)** Diverse club regions (red nodes) of HC. **(E)** An illustrated diagram of the three levels of connections: hub connections, linking two hub nodes (rich-club or diverse-club nodes); feeder connections, linking one hub node to one peripheral node; and local connection, linking two peripheral nodes.

The top 13 (15%) highest-degree nodes, based on the nodal degree in the HC group, represented rich-club regions. The selection of rich-club regions ensured equal numbers of nodes in the functional network analyses and was beneficial to analyze the disruption of rich-club organization in the SCD and aMCI groups. The rich-club regions included the left and right postcentral, the left and right insula (INS), the left and right superior temporal gyrus (STG), the left and right rolandic operculum (ROL), the left and right precentral, the left and right anterior cingulate and paracingulate gyri (ACG), and the left caudate. The remaining regions were defined as peripheral regions (see Figure 1).

**FIGURE 2:**
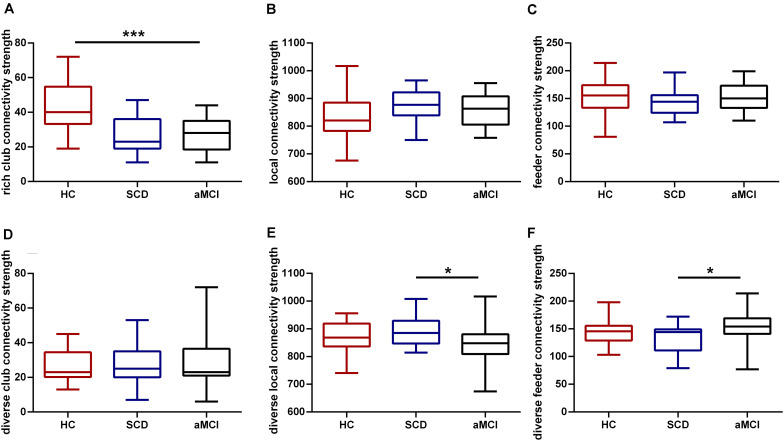
Rich-club and diverse-club organizations during disease progression. Group differences in rich-club and diverse-club network properties at S = 15% are exhibited. Boxplots exhibit the dispersion of age- and sex-adjusted connectivity strengths for **(A)** rich club, **(B)** feeder, **(C)** local, **(D)** diverse club, **(E)** diverse feeder, **(F)** diverse local. *p < 0.05, ***p < 0.001. Bonferroni correction was used for the two-sample *t*-test.

Among the three groups, a significant difference (*F* = 15.869, *p* < 0.001) was observed in the rich-club connectivity strength. Compared with the HC, aMCI showed significant decreases (*p* < 0.001) in rich-club connectivity strength (*p* < 0.05, Bonferroni-corrected). There was no difference between the SCD and HC groups in rich-club connection strength, but there is a downward trend. All the results were obtained after controlling for the effects of age and sex (see Figure 2).

The top 13 (15%) highest participate coefficient nodes were chosen as diverse-club regions, including the left and right temporal pole (middle temporal gyrus, TPOmid), the right temporal pole (superior temporal gyrus, TPOsup), the left ROL, the left ACG, the left INS, the right caudate, the right middle temporal gyrus (MTG), the right STG, the right orbital part of inferior frontal gyrus (ORBinf), the left inferior parietal gyrus (IPL), the left supramarginal gyrus (SMG), and the left olfactory cortex (OLF) (see Figure 1).

Significant group differences in three groups were found in feeder (*F* = 3.970, *p* = 0.023) and local (*F* = 4.015, *p* = 0.022) connectivity strength in diverse club after controlling for the effect of age and sex. Compared with the SCD, the aMCI group showed lower feeder diverse (*p* = 0.021) and local diverse (*p* = 0.019) connectivity strength (*p* < 0.05, Bonferroni-corrected) (see Figure 2).

### Disrupted Node Metrics of Overlapping Nodes

**FIGURE 3:**
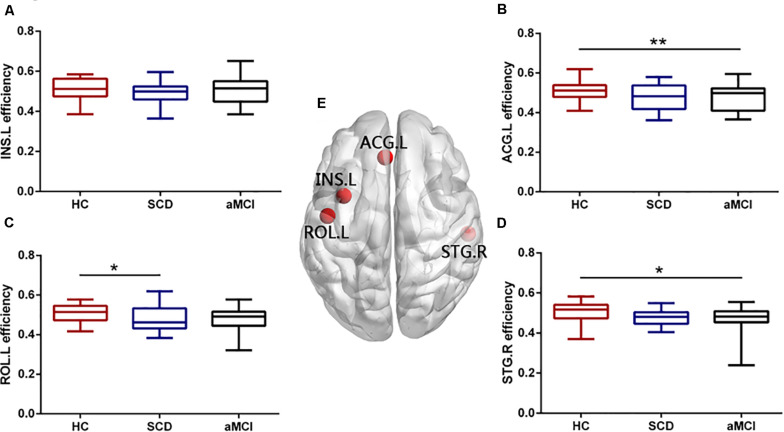
Group differences in nodal efficiency of overlapping nodes during disease progression. Boxplots exhibit the dispersion of nodal efficiency metrics for the age- and sex-adjusted of **(A)** INS.L, **(B)** ACG.L, **(C)** ROL.L, and **(D)** STG.R. **(E)** Four overlapping nodes, including INS.L, ACG.L, ROL.L, and STG.R. *p < 0.05, **p < 0.01. INS.L, the left insula; ACG.L, the left anterior cingulate and paracingulate gyri; ROL.L, the left rolandic operculum; STG.R, the right superior temporal gyrus.

**FIGURE 4:**
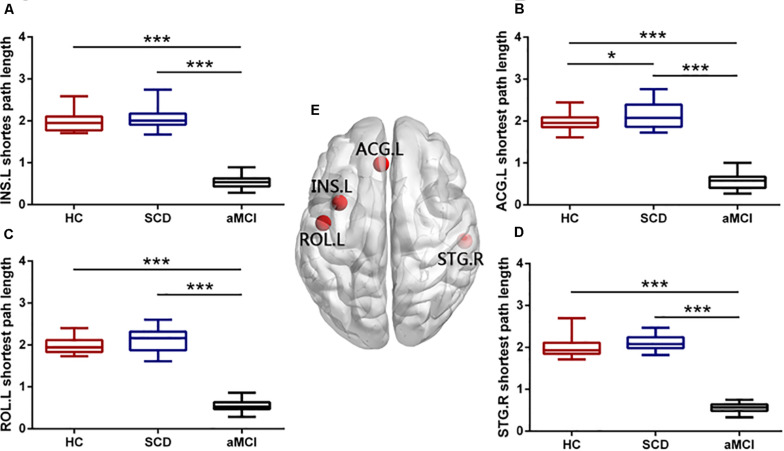
Group differences in nodal shortest path length of overlapping nodes during disease progression. Boxplots exhibit the dispersion of shortest path length metrics for the age- and sex-adjusted of (**A**) INS.L, **(B)** ACG.L, **(C)** ROL.L, and **(D)** STG.R. **(E)** Four overlapping nodes, including INS.L, ACG.L, ROL.L, and STG.R. *p < 0.05, ***p < 0.001. INS.L, the left insula; ACG.L, the left anterior cingulate and paracingulate gyri; ROL.L, the left rolandic operculum; STG.R, the right superior temporal gyrus.

We found that there were four nodes, both with high node degree and participation coefficient, including the left INS (INS.L), the right STG (STG.R), the left ROL (ROL.L), and the left ACG (ACG.L). The ANOVA showed the group difference in the nodal efficiency of the ROL.L (*F* = 3.294, *p* = 0.043), the ACG.L (*F* = 6.235, *p* = 0.003), and the STG.R (*F* = 3.885, *p* = 0.025) (see Figure 3). Significant declines were viewed in the nodal efficiency of ACG.L (*p* = 0.003) and STG.R (*p* = 0.034) in the aMCI group versus the HC group and the nodal efficiency of INS.L (*p* = 0.049) in the SCD group versus the HC group. There was no significant difference in the efficiency of the INS.L between any two of the three groups (*p* < 0.05, Bonferroni-corrected). Group difference (ANOVA) of nodal shortest path length was found in all the overlapping nodes, including ROL.L, INS.L, ACG.L, and STG.R (see Figure 4). Compared with the HC, the aMCI group showed significantly declined nodal shortest path length in the four overlapping nodes (all *p* < 0.001), whereas the SCD group showed increased nodal shortest path length in the ACG.L (*p* = 0.017). Compared with the SCD, all the nodal shortest path length of four overlapping nodes showed similar significantly decreased in the aMCI group (all *p* < 0.001). All the results of two-sample tests were calculated after controlling the effect of age and sex (*p* < 0.05, Bonferroni-corrected). There was no significant difference in betweenness centrality, nodal clustering coefficient, and node degree between the three groups.

**FIGURE 5:**
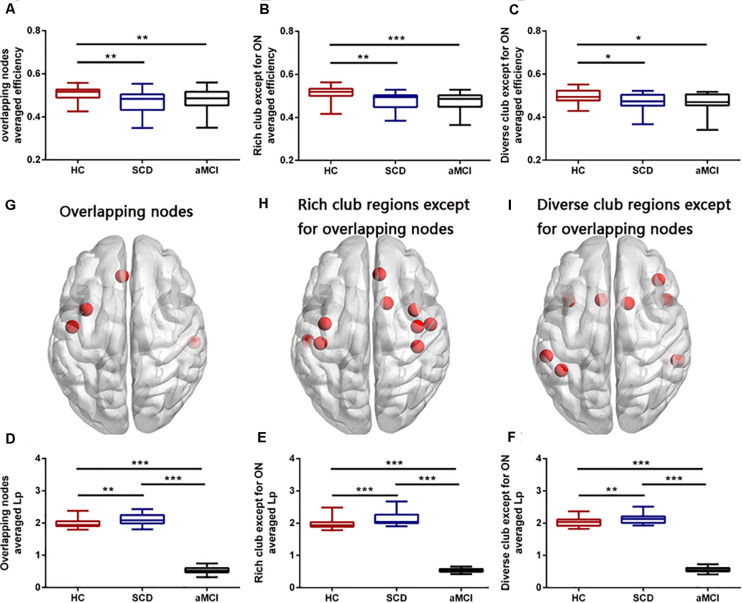
Group differences in averaged efficiency and averaged *L*_p_ of overlapping nodes, rich-club regions except for overlapping nodes, and diverse club except for overlapping nodes. Boxplots exhibit the dispersion of averaged nodal efficiency and nodal shortest path length for the age- and sex-adjusted. **(A,D)** Average nodal efficiency and shortest path length in overlapping nodes. **(B,E)** Averaged nodal efficiency and shortest path length in rich-club regions except for overlapping nodes. **(C,F)** Averaged nodal efficiency and shortest path length in diverse-club regions except for overlapping nodes. **(G–I)** Brain regions showing the four overlapping nodes, rich-club regions except for overlapping nodes, and diverse-club regions except for overlapping nodes. *p < 0.05, **p < 0.01, ***p < 0.001. ON, overlapping nodes; L_p_, nodal shortest path length.

Group differences were found both in the averaged nodal efficiency and nodal shortest path length of all the four overlapping nodes (*F* = 7.699, *p* = 0.001; *F* = 952.574, *p* < 0.001), rich club except for overlapping nodes (*F* = 12.991, *p* < 0.001; *F* = 938.875, *p* < 0.001), and diverse club except for overlapping nodes (*F* = 4.542, *p* = 0.014; *F* = 1141.592, *p* < 0.001) (see Figure 5). Compared with the HC, both the SCD and aMCI groups showed significant declined averaged nodal efficiency in overlapping nodes (*p* = 0.009; *p* = 0.002), the rich club except for overlapping nodes (*p* = 0.001; *p* < 0.001), and the diverse club except for overlapping nodes (*p* = 0.027; *p* = 0.046). In addition, compared with the HC, the SCD group showed significantly higher averaged nodal shortest path length in overlapping nodes (*p* = 0.009), the rich club except for overlapping nodes (*p* < 0.001), and the diverse club except for overlapping nodes (*p* = 0.009), whereas the aMCI group showed significantly lower averaged nodal shortest path length in overlapping nodes (*p* < 0.001), the rich club except for overlapping nodes (*p* < 0.001), and the diverse club except for overlapping nodes (*p* < 0.001). Compared with the SCD, the aMCI group showed significantly decreased averaged nodal shortest path length in overlapping nodes (*p* < 0.001), the rich club except for overlapping nodes (*p* < 0.001), and the diverse club except for overlapping nodes (*p* < 0.001).

### Behavioral Significance of the Disrupted Network and Nodes Metrics

**FIGURE 6:**
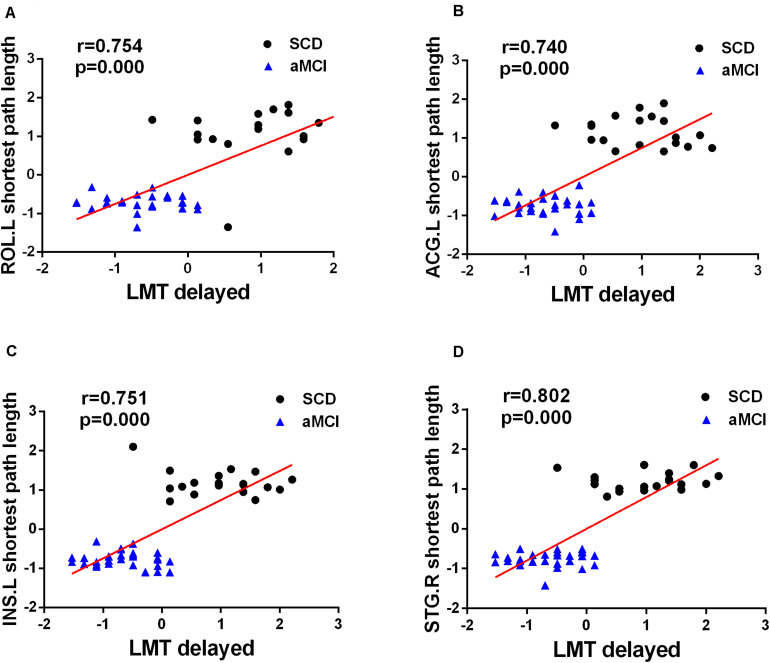
Significant associations between altered network metrics and cognitive function. Age and sex were used as covariates of results (Bonferroni-corrected, *p* < 0.05). LMT-delayed, the Wechsler Logical Memory delayed recall.

A correlation analysis was performed between changed network and nodal metrics and cognitive scales, including MMSE, LMT-immediate, and LMT-delayed (Bonferroni-corrected, *p* < 0.05). The analysis showed that the nodal shortest path length of four overlapping nodes, including ROL.L (*R* = 0.754, *p* < 0.001), ACG.L (*R* = 0.740, *p* < 0.001), INS.L (*R* = 0.751, *p* < 0.001), and STG.R (*R* = 0.802, *p* < 0.001), were positive correlation with LMT-delayed after controlling the effects of age and sex (see Figure 6).

### Hippocampal Volume

**FIGURE 7:**
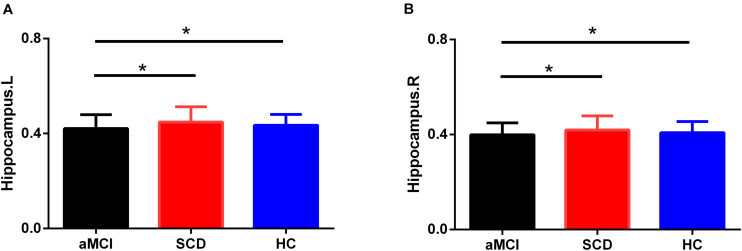
Group differences in averaged bilateral hippocampus volumes during disease progression. Bar charts exhibit the dispersion of age- and sex-adjusted connectivity strengths for **(A)** left hippocampus, **(B)** right hippocampus. Bonferroni correction was used for the two-sample *t*-test. L, left; R, right.

Group differences were found in the right hippocampus (*F* = 5.806, *p* = 0.005) and left hippocampus (*F* = 5.201, *p* = 0.008) of the three groups, including aMCI, SCD, and HC. The results showed that the aMCI group showed a significantly decreased volume of the bilateral hippocampus compared with the HC and SCD after controlling the effects of age and sex (Bonferroni-corrected, *p* < 0.05) (see Figure 7).

## Discussion

The purpose of the current study was to investigate changes in network metrics and rich-club and diverse-club organizations across the preclinical AD spectrum, including SCD and aMCI, and explore how the changed functional network affected cognitive function. Consistent with our assumption, our research had three main findings. Firstly, the network properties, rich club, and diverse club had different degrees of disruption in the SCD and aMCI groups. Secondly, the overlapping nodes of the rich-club and diverse-club regions in the SCD and aMCI groups showed significantly altered nodal metrics, which might reflect the progress of the AD spectrum. Finally, the altered shortest path length of four overlapping nodes between the rich-club and diverse-club regions was associated with impaired cognitive function.

### Global Network Disruption Across Three Groups

Our results exhibited that the functional networks of the SCD and aMCI patients also showed a small-world topology, which accords with previous studies (Sun et al., 2014, 2018). Compared with the HC, the aMCI showed significantly lower small-worldness, lower normalized clustering coefficient, and higher characteristic path length. Although there was no significant difference between the SCD group and HC group, the SCD group also had the same trend as the aMCI group in the small-world parameters (σ), normalized clustering coefficient (γ), and characteristic path length (*L*_p_). Jacini et al. (2018) applied functional connectivity to MEG data and found no global topological changes in aMCI, which was not consistent with our results. The inconsistent results may be due to patient heterogeneity or the nature that rs-fMRI and diffusion tensor imaging were more sensitive to capture changes of the global network than MEG (Jacini et al., 2018). The small-world networks were featured by a high representation of strongly interconnected networks, equipped with higher γ and lower *L*_p_ (Wang et al., 2013). The clustering coefficient represented local information transfer’s efficiency for specialized processing, and the short characteristic path length represented a high level of global communication efficiency (Van Den Heuvel et al., 2008; Li et al., 2016). The altered σ, γ, and L_p_ in the SCD and aMCI groups indicated the disturbed balance between local specialization and global integration (Sun et al., 2014). Moreover, the aMCI group showed a significant decline in global efficiency compared with the HC, which was consistent with previous studies, also proving the potential mechanisms of disconnection in the AD spectrum (Sun et al., 2018). More severe disruptions of network metrics were found in the aMCI patients relative to the SCD patients. Compared with the traditional signal network studies, brain functional network researches provided a systematic and comprehensive view to survey the preclinical AD spectrum progression.

### Rich-Club and Diverse-Club Organizations Disruption Across Three Groups

The general tendency of three type connections of rich club and diverse club was similar in the preclinical AD spectrum progression. Note that the aMCI group showed significantly decreased rich-club connection, whereas the feeder and local connection stayed relatively stable compared with the HC. The phenomenon was in accord with the previous research that hubs appear to be preferentially affected in AD patients (Shu et al., 2018). The rich club played a key role in global information transmission and may provide important information on how the AD spectrum affected brain functional network (Albert et al., 2000; Yan et al., 2018). The possible reason why rich-club regions seemed to be vulnerably affected were the higher probability of damage that accompanied stronger connections and greater susceptibility to oxidative and metabolic stresses (Fornito et al., 2015; Shu et al., 2018).

Recently, several pieces of research suggested that using the participation coefficients to define the hub regions will provide a new perspective on brain network analysis (Guimera and Nunes Amaral, 2005b; Power et al., 2013; Bertolero et al., 2017). The participation coefficient evaluates the distribution of the edges among a network’s communities (Power et al., 2013). If nodes edges are entirely restricted to its community, its participation coefficient is zero. When the edges of a node are dispersed among all communities, the participation coefficient of the node is one, which is the maximal value. A node with higher participation coefficients is called “connector” hub (Power et al., 2013). Recent researches have referred to nodes with high participation coefficients as a diverse club (Bertolero et al., 2017). In the present study, the aMCI patients showed increased diverse feeder connection and decreased diverse local connection compared with the SCD group. There was no significant difference in the diverse-club connection across the three groups, which might suggest that the diverse club is more stable than the rich club in the progression of AD (Bertolero et al., 2017). This may be because the diverse club showed more highly interconnected and exhibits stronger clubness than the rich club (Bertolero et al., 2017). In the early stage of the AD spectrum, the stability of diverse clubs can maintain relatively normal information integration and coordination between the networks. The investigation of the rich-club and diverse-club organizations revealed distinct roles in the functional network and will facilitate to identify the SCD and aMCI groups.

### Node Metrics Disruption of Overlapping Nodes Across Three Groups

The overlapping nodes between rich-club regions and diverse-club regions included INS.L, STG.R, ROL.L, and ACG.L. Because they had the highest node degree and the highest participation coefficient in the meantime, the disorder of these nodes might be the main reason of abnormal communication in the brain. Studying the changes in these node metrics may provide a potential biomarker for the diagnosis of the AD spectrum and facilitate the understanding of the progressive change in clinical manifestation of the AD spectrum.

In the current study, there was no significant difference in the nodal efficiency of INS.L among the three groups, including SCD, aMCI, and HC. The node efficiency of INS.L and ROL.L showed a similar trend of decreasing before increasing. Many historians have argued that the insula, as limbic integration cortex, had strong connections with the primary olfactory cortex (Augustine, 1996; Bonthius et al., 2005). A number of studies have found that dysosmia may be one of the earliest symptoms of the AD spectrum and olfaction was significantly impaired in AD (Bonthius et al., 2005). In consequence, the insula could be the key cortical marker for olfactory disorders in AD. Moreover, the INS.L was thought to be important in the maintenance of memory performance in the early stage of the AD-related pathology (Lin et al., 2017). Insula pathology may be an important cause of the increased incidence of heart failure, bronchopneumonia, and other life-threatening visceral dysfunction in the course of the AD spectrum (Bonthius et al., 2005). The stabilization of INS.L might be a compensation mechanism to fight against the declined cognitive. Compared with the HC, the SCD showed decreased nodal efficiency in ROL.L, which might mean that in the evolution of the disease, the ROL.L was a priority to be affected. The operculum, adjacent to the insula, was responsible for sensory, motor, autonomic, and cognitive processing (Maliia et al., 2018). The ROL was a major region involved in the language processing system, which played an important role in a variety of neurologic and psychiatric conditions (Maliia et al., 2018; Shan et al., 2018; Zhan et al., 2019). The proximity of the anatomical structures may be the reason why they have a similar tendency to change in the progression of the AD spectrum. However, there has been no previous research on the role of ROL.L in the AD spectrum, and this may provide a new perspective for future research.

In the present study, the aMCI showed decreased nodal efficiency of both ACG.L and STG.R compared with the HC. According to previous researches, ACG.L and STG.R played important roles in the AD spectrum. Previous studies have reported that AD associated with tau neurofibrillary tangles and hypermethylation; in the meantime, increased DNA methylation and tau lesions were found in the STG of AD brain (Smith et al., 2016; Watson et al., 2016; Kaufman et al., 2018). Recent research found that the presynaptic vesicle protein CSPalpha played a vital role in synaptic degeneration and protection in AD, whereas CSPalpha expression was decreased in the hippocampus and STG in the progress of AD. All the studies proved that STG was necessary for the pathology of AD. A more important fact is that STG was thought to be involved in emotional processing and social cognition and play a key role in the depressive symptoms of AD patients (Arnsten and Rubia, 2012). However, the correlation results showed that there was no significant correlation between altered nodal metrics and GDS. The reason might be the small sample in the current study. Furthermore, ACG.L, belonging to the executive control network, participated in regulating emotional and cognitive behavior, which has shown decreased functional connectivity in AD (Zhao et al., 2019). Previous longitudinal research of brain metabolic alterations from aMCI to AD showed that aMCI converters had a significant metabolic decline in the left ACG than aMCI non-converters (Fouquet et al., 2009). Node efficiency can represent the ability of a node to transmit information to other nodes. When nodes were damaged in the process of AD spectrum progression, node efficiency will change with it. According to the present results, the nodal efficiency of the ACG.L and STG.R can be a hallmark of identifying the aMCI group.

The nodal shortest path length of the four overlapping nodes (INS.L, STG.R, ROL.L, and ACG.L) followed remarkably similar change patterns with the AD spectrum that progressed. Compared with the HC, the SCD group showed an increasing trend, whereas the aMCI showed a significant decreasing trend in nodal shortest path length. There was a significant difference between the aMCI and SCD, which can help to distinguish the different stages of the preclinical AD spectrum. It is worth noting that only the ACG.L showed significantly increased nodal shortest path length in the SCD group compared with the HC. To explore how these overlapping nodes, rich-club regions except for overlapping nodes, and diverse-club regions except for overlapping nodes evolved in the progress of the AD spectrum, we calculated their respective average node efficiency and nodal shortest path length. The result showed that the progression of three types of nodes was consistent. It showed that the nodal efficiency of hub regions decreased progressively as the disease progresses, whereas the nodal shortest path length ascends in the SCD group and then descends in the aMCI group. As a result, the increased network communication efficiency in the hub regions of the aMCI patients might be compensated for the decreased global efficiency.

### Behavioral Significance of Altered Nodal Metrics Across Three Groups

The current study showed observably positive correlations between nodal shortest path length of four overlapping nodes and LMT-delayed in patients with SCD and aMCI. LMT-delayed was used to evaluate long-term verbal memory performance. In the present study, the results showed that there was no significant difference between the SCD and HC in LMT-delayed adjusted for age and sex. Although the cognitive changes of the SCD group were subtle and with no clinical significance, the correlation between the node metrics of four overlapping nodes and LMT-delayed in the SCD and aMCI groups predicted that potential deterioration in the overlapping nodes might be present before clinical diagnosis. Abnormal clinical memory function may be attributed to the abnormal graph theory index of the four overlapping nodes. The results were consistent with previous reports that the four overlapping nodes, including INS.L, ACG.L, ROL.L, and STG.R, were associated with memory and cognitive performance (Arnsten and Rubia, 2012; Maliia et al., 2018; Dimond et al., 2019). Notably, the four overlapping nodes, both with high participation coefficient and high node degree, were core nodes of network information exchange and integration and the absolute center of the network. This further demonstrated that the changing nodal metrics of the four overlapping nodes might reveal the pathology of the AD spectrum.

### Hippocampal Volume Analysis

In the present study, compared with the HC and SCD groups, significantly decreased bilateral hippocampal volumes were observed in the aMCI group. Notably, there was no difference in bilateral hippocampal volumes between the HC and SCD groups. Actually, the results of hippocampal volume atrophy in patients with SCD were inconsistent (Striepens et al., 2010; Stewart et al., 2011; Ryu et al., 2017; Van Rooden et al., 2018; Caillaud et al., 2020). Some studies observed smaller hippocampal volumes in SCD subjects, whereas others did not. The inconsistencies in results may be due to different observation time points and disease stage. Furthermore, loss of hippocampal volume may contribute to SCD, whereas having SCD is not *per se* associated with a smaller brain or hippocampal volume (Blom et al., 2019). Importantly, a large number of neuroimaging studies proved that alterations in the functional connectivity were thought to precede symptoms and structural changes in SCD by several years (Bateman et al., 2012; Hayes et al., 2017). Especially, the present study also showed altered connectivity strength and nodal attributes in the SCD group. This is why researches in recent years have focused on the change of resting-state network, hoping to find more accurate biomarkers of the AD spectrum and predict the probability of AD in the early stage.

### Limitations

The current study had two major limitations. Firstly, the MRI data were from the ADNI database, and the sample size was small, which might hinder us from detecting small effect values and result in some null results. Secondly, there were only four cognitive scales in the present study, so some underlying information and correlativity cannot be disclosed. Our research team has been recruiting volunteers for the study and conducting detailed neuropsychological tests to further verify the present results.

## Conclusion

By using the rs-fMRI combined with graph analysis, our study demonstrated disrupted topologic organization of brain functional connectome in the SCD and aMCI groups relative to the HC. The three types of connections of the rich club and the diverse club might be contributed to the diagnosis of the aMCI group and the identification of the SCD and aMCI groups. Additionally, the nodal efficiency and nodal shortest path length of the overlapping nodes among rich and diverse clubs showed significant differences between groups, which might be important biomarkers of AD spectrum diagnosis. In a word, the analysis of the brain functional network might provide potential biomarkers for the early detection of episodic memory decline in elderly individuals.

## Data Availability Statement

The datasets presented in this study can be found in online repositories. The names of the repository/repositories and accession number(s) can be found below: http://adni.loni.usc.edu/.

## Ethics Statement

The studies involving human participants were reviewed and approved by Declaration of Helsinki promulgated by the National Institute of Health. The patients/participants provided their written informed consent to participate in this study.

## Author Contributions

CXu, HS, CXi, and JC designed the study. CXu, HS, CXi, JC, GH, WQ, YY, JR, and WY collected the data. CXu and HS analyzed the data and prepared the manuscript. All the authors contributed to the article and approved the submitted version.

## Conflict of Interest

The authors declare that the research was conducted in the absence of any commercial or financial relationships that could be construed as a potential conflict of interest.

## Footnotes

1 adni.loni.usc.edu

2 http://www.mathworks.com/products/matlab/

3 http://restfmri.net/forum/RESTplusV1.2

4 https://www.fil.ion.ucl.ac.uk/spm/

5 http://www.nitrc.org/projects/gtetna/

6 http://www.spss.com/

7 https://www.nitrc.org/projects/wfu_pickatlas/
