# Supplementary material for: Disrupted Patterns of Rich-Club and Diverse-Club Organizations in Subjective Cognitive Decline and Amnestic Mild Cognitive Impairment
Source: Front Neurosci. 2020 Oct 15;14:575652. doi: 10.3389/fnins.2020.575652 (PMC7593791; doi:10.3389/fnins.2020.575652)
Supplement: Supplementary file 1 [file Data_Sheet_1.doc]

***SI Methods***

**Participants**

The diagnosis of SCD was made using the standard criteria describe in the ADNI-2 procedures manual: 1) The Cognitive Change Index (CCI: total score from frist12 items > 16) was used to assess the subjective memory concerns of SCD participates; 2) normal cognitive performance on the Mini-Mental State Exam (MMSE), the Wechsler Logical Memory Immediate (LMT) and LMT Delayed Recall (LMT-Delayed). Furthermore, the selected aMCI patients were based on the ADNI-2 procedures manual: 1) memory complaint; 2) Abnormal memory function documented by scoring within the education adjusted ranges on the Logical Memory II subscale (Delayed Paragraph Recall, Paragraph A only) from the Wechsler Memory Scale Revised (≤8 for 16 or more years of education; ≤4 for 8-15 years of education; ≤2 for 0-7 years of education); 3) Clinical Dementia Rating (CDR) = 0.5. Memory Box score must be at least 0.5; 4) MMSE scores between 24 and 30; 5) mo dementia and no signal of depression (Geriatric Depression Scale, GDS < 6). Additionally, the criteria of HC were: 1) no memory complaints; 2) normal cognitive performance, MMSE between 24 and 30, and GDS < 6; 3) CDR=0.

**MRI scanning**

The structure images were obtained by 3D magnetization-prepared rapid gradient-echo (MPRAGE) T1-weighted sequence. The parameters were: repetition time (TR) = 2300ms; echo time (TE) = 2.98ms; inversion time (TI) = 900ms; number of slices = 170, thickness = 1.2mm, field of view (FOV) = 256 mm ×240 mm; voxel size = 1.1 × 1.1 × 1.2mm^3^; flip angle (FA) = 9^0^; bandwidth = 240Hz/pix.

The rsfMRI images were acquired using the gradient-echo echo-planar imaging (GRE-EPI) sequence with 140 volumes. The parameters were: TR = 3000ms; TE = 30ms; number of slices = 48; slice thickness = 3.3mm; matrix = 64 × 64; FA = 80^0^; spatial resolution = 3.31 × 3.31 × 3.31mm^3^.

**Image preprocessing**

The first 5 volumes were discarded to reduce the possible instability of MRI signal. The remaining 135 volumes were corrected for the intra-volume acquisition time differences among slices and head motion (six-parameter rigid body) effects during the scan. Participants with excessive head motion (cumulative translation or rotation > 2.0mm or 2.0^0^) were discarded (Chen et al., 2016b;Chen et al., 2019b). Subsequently, the T1-weight images were co-registered to the mean rsfMRI image and we chose affine regularization in segmentation with European. The fMRI images were then spatially normalized to the Montreal Neurological Institute (MNI) stereotactic space and resampled to an isotropic voxel size of 3mm. Next, spatial smoothing with a 6-mm full-width half-maximum Gaussian kernel and detrending were used to reduce spatial noise and the difference of anatomical structures (Chen et al., 2016c). Finally, we chose nuisance covariate regression with 6 motion parameters, global signal (Chen et al., 2016a;Chen et al., 2019a), white matter signal, and cerebrospinal fluid signal and filter at 0.01-0.08Hz.

**Definitions of network metrics**

For the global network metrics, we quantified the characteristic path length (L_p_), normalized characteristic path length (λ), clustering coefficient (C_p_), normalized clustering coefficient (γ), small-world parameters (σ), global efficiency (E_g_), and local efficiency (E_loc_). For the regional characteristics, we considered the nodal clustering coefficient, nodal shortest path length, and betweenness centrality. All network analyses were performed using the GRETNA software (<http://www.nitrc.org/projects/gretna/>). Detailed definitions of the network metrics and nodal metrics are provided as follows.

**Network efficiency**. The global efficiency of G measures the global efficiency of the parallel information transfer in the network, which can be computed as:


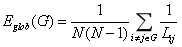


Where L_ij_ is the shortest path length between node i and node j in G.

The local efficiency of G shows how efficient the communication is among the first neighbors of the node i when it is removes and reveals how much the network is fault tolerant (Achard et al., 2007). The local efficiency of a graph is defined as:


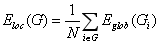


Where G_i_ denotes the subgraph composed of the nearest neighbors of node i.

**Small-world properties**. Small-world network parameters mainly include characteristic path length (L_p_), normalized characteristic path length (λ), clustering coefficient (C_p_), normalized clustering coefficient (γ), and small-world parameters (σ). In the study, we investigated the small-world properties of the binary brain networks.

The path length between node i and node j is defined as the sum of the edge lengths along the path. The shortest characteristic path length plays an important role in the information transmission and communication of network. The shortest path length, L_ij_, is defined as the length of the path for node i and node j with the shortest length. The shortest path length was computed as follows:


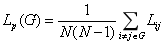


Where N is the number of nodes in the network.

The clustering coefficient of a node i, C(i), which was defined as the possibility whether the neighborhoods were connected with each other or not, was computed as follows:


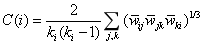


where k_i_ is the degree of node i and
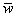
 is the weight of edge, which is scaled by the maximum weight of the network. The clustering coefficient, C_p_, of the network is the average of the clustering coefficient over all nodes and indicates the degree of local interconnection or clustering in the network. If the nodes are isolated or have just one connection, i.e., k_i_ = 0 or k_i_ = 1, the clustering coefficient is zero.

To examine the small-world properties, the characteristic path length (L_p_), and clustering coefficient (C_p_) of the networks were compared with those of random networks. In the present study, we generated 1000 matched random network with the same number of nodes, edges, and degree distribution as the real networks. Furthermore, we computed the normalized characteristic path length,
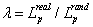
 and the normalized clustering coefficient,
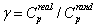
, where
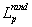
 and
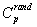
are the averaged L_p_ and the averaged C_p_ of 1000 matched random networks. If the studied network has a high clustering coefficient (
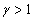
) and short characteristic path length (
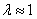
) relative to the random network, then the network has the property of “small world” (Watts et al., 1998). Humphries et.al combined these two measurements (γ and λ) into a simple quantitative metric, small-wordness,
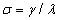
, to measure the “small world” property (Humphries et al., 2006). When σ >1, the network has the “small world” property.

**Regional nodal characteristics**. To determine the nodal characteristics of the brain networks, we computed the nodal degree, nodal efficiency, nodal clustering coefficient, nodal shortest path length, and betweenness centrality.

The nodal degree, k_nodal_, defined as the number of edges connected to the node, is a fundamental basic network measure to assess the central role of a region in brain networks. A node with a great nodal degree is classified as a hub that is highly connected to other nodes. The nodal degree was computed as follows:


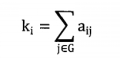


where a_ij_ means there is a directly connection between node i and node j in G.

The nodal efficiency, E_nodal_, is defined as:


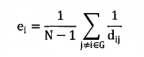


where d_ij_ is the shortest path length between node i and node j in G. Node efficiency reflects the ability of a node to transmit information to other nodes in the network.

The betweenness centrality, B_nodal_, is defined as:


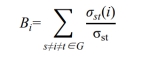


where
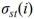
is the number of shortest path length from node s to node t. The betweenness centrality measures the importance of node i in G as a bridge.

**References:**

Achard, S., and Bullmore, E. (2007). Efficiency and cost of economical brain functional networks. *PLoS Comput Biol* 3**,** e17. doi:10.1371/journal.pcbi.0030017

Chen, J., Chen, G., Shu, H., Chen, G., Ward, B.D., Wang, Z., et al. Alzheimer's Disease Neuroimaging, I. (2019a). Predicting progression from mild cognitive impairment to Alzheimer's disease on an individual subject basis by applying the CARE index across different independent cohorts. *Aging (Albany NY)* 11**,** 2185-2201. doi:10.18632/aging.101883

Chen, J., Duan, X., Shu, H., Wang, Z., Long, Z., Liu, D., et al. Zhang, Z. (2016a). Differential contributions of subregions of medial temporal lobe to memory system in amnestic mild cognitive impairment: insights from fMRI study. *Sci Rep* 6**,** 26148. doi:10.1038/srep26148

Chen, J., Shu, H., Wang, Z., Liu, D., Shi, Y., Xu, L., and Zhang, Z. (2016b). Protective effect of APOE epsilon 2 on intrinsic functional connectivity of the entorhinal cortex is associated with better episodic memory in elderly individuals with risk factors for Alzheimer's disease. *Oncotarget* 7**,** 58789-58801. doi:10.18632/oncotarget.11289

Chen, J., Shu, H., Wang, Z., Zhan, Y., Liu, D., Liao, W., et al. Zhang, Z. (2016c). Convergent and divergent intranetwork and internetwork connectivity patterns in patients with remitted late-life depression and amnestic mild cognitive impairment. *Cortex* 83**,** 194-211. doi:10.1016/j.cortex.2016.08.001

Chen, J., Shu, H., Wang, Z., Zhan, Y., Liu, D., Liu, Y., and Zhang, Z. (2019b). Intrinsic connectivity identifies the sensory-motor network as a main cross-network between remitted late-life depression- and amnestic mild cognitive impairment-targeted networks. *Brain Imaging Behav*. doi:10.1007/s11682-019-00098-4

Humphries, M.D., Gurney, K., and Prescott, T.J. (2006). The brainstem reticular formation is a small-world, not scale-free, network. *Proc Biol Sci* 273**,** 503-511. doi:10.1098/rspb.2005.3354

Watts, D.J., and Strogatz, S.H. (1998). Collective dynamics of 'small-world' networks. *Nature* 393**,** 440-442. doi:10.1038/30918
